# OptiSpot: minimizing application deployment cost using spot cloud resources

**DOI:** 10.1007/s10586-016-0568-7

**Published:** 2016-04-23

**Authors:** Daniel J. Dubois, Giuliano Casale

**Affiliations:** Department of Computing, Imperial College London, London, UK

**Keywords:** Cloud provisioning, Application deployment, Fluid-approximated queueing networks, Spot cloud, Random environment, Bidding strategy

## Abstract

The spot instance model is a virtual machine pricing scheme in which some resources of cloud providers are offered to the highest bidder. This leads to the formation of a spot price, whose fluctuations can determine customers to be overbid by other users and lose the virtual machine they rented. In this paper we propose OptiSpot, a heuristic to automate application deployment decisions on cloud providers that offer the spot pricing model. In particular, with our approach it is possible to determine: (i) which and how many resources to rent in order to run a cloud application, (ii) how to map the application components to the rented resources, and (iii) what spot price bids to use to minimize the total cost while maintaining an acceptable level of performance. To drive the decision making, our algorithm combines a multi-class queueing network model of the application with a Markov model that describes the stochastic evolution of the spot price and its influence on virtual machine reliability. We show, using a model developed for a real enterprise application and historical traces of the Amazon EC2 spot instance prices, that our heuristic finds low cost solutions that indeed guarantee the required levels of performance. The performance of our heuristic method is compared to that of nonlinear programming and shown to markedly accelerate the finding of low-cost optimal solutions.

## Introduction

Cloud computing is a popular paradigm for offering compute capacity as a service. In particular, the cloud gives flexibility to decide and modify the speed, the number, and the lease time of virtual machines (VMs). There are several pricing strategies for renting VMs, among which are often mentioned two categories: *on-demand pricing* and *spot pricing*. On-demand pricing guarantees that a resource is available for a fixed price, which is proportional to the time the resource is rented. In spot pricing, instead, resources are offered at a variable price, called the spot price, which is arbitrarily decided by the cloud provider. Spot pricing requires users to bid a maximum price they are willing to pay for. If the bid price is greater than the current spot price, the virtual machine will be charged at the spot price. However, if the spot price exceeds the bid price, the VM will receive a termination notice and eventually be reclaimed by the provider. The advantage of spot instances is that their price tends to be lower than the on-demand price most of the time, but from time to time, when the cloud provider has a shortage of resources, it can temporarily make the spot price steep (much higher than the on-demand price) in order to have most of spot resources back. This makes the decision of choosing a bid price both difficult and important. While a number of works have considered this problem in recent years [9, 17, 29, 31], the problem of deciding bid prices in light of performance requirements or constraints on the application architecture is more complex and still poorly understood.

This paper, which extends [11], aims at helping cloud users to take maximum advantage from spot instances by supporting the following decisions:

- What type of virtual resources should be rented for a given application?
- How to efficiently map the components of an application (e.g., web server VMs, a database VMs) to the rented resources?
- What is the optimal bid price for each resource that allows to fulfill quality of service requirements?

Specifically, we focus on applications developed according to the model-driven engineering approach, in which a performance model of the application can be automatically generated through model-to-model transformations. For example, queueing networks can be automatically generated from UML or Palladio Component Model diagrams [3, 22]. The problem of executing the decisions, such as concretely migrating the virtual resources is out of the scope of this paper, which focuses on the decision problem.

The main technical innovations of this paper are as follows:

- a heuristic, called OptiSpot, to jointly solve the bidding and allocation problem, which are in general NP-hard;
- what is, to our knowledge, the first application in the area of bidding of extended queueing network models that include a model of the operational environment. The latter, which is referred to as *random environment* model [6], captures the stochastic nature of the operational environment, in which VMs can be lost and restarted as a result of spot price fluctuations and the consequent temporary switch to an on-demand pricing model.
- the use of advanced fluid analysis techniques to accurately approximate response time percentiles, which are commonly used to constraint performance in service-level agreements, but which are usually hard to compute in queueing networks. Compared to more complex approximations for accurate percentile assessment, such as Laplace transforms, this method is fast enough for run-time application.

OptiSpot can quickly find a local optimal solution. We validate the accuracy of this solution by considering the queueing network model of a real enterprise resource planning (ERP) application and real recent historical data of Amazon EC2 spot prices. We compare our results with an approach that uses a nonlinear optimization algorithm and show that our heuristics provides better results in less time.

The rest of this paper is organized as follows. Section 2 gives a motivating example. Section 3 discusses the problem statement and defines the reference model. Section 4 presents the OptiSpot heuristic to provision and map application components to cloud resources. Section 5 describes the bidding price strategy we used in our case study and how it can be represented as a random environment. Our approach is later evaluated in Sects. 6 and 7. Section 8 surveys related work. Lastly, Sect. 9 concludes the paper and outlines possible extensions.

**Fig. 1:**
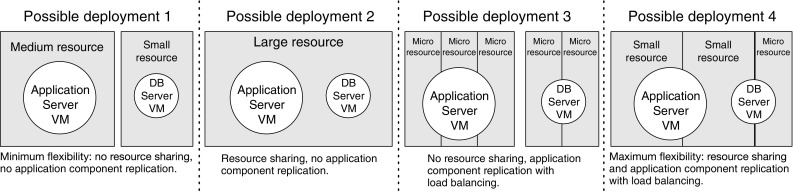
Different strategies for deploying the application components of the SAP ERP application to cloud resources. *Circles* represent applications, their size indicates their ECU requirements. *Rectangles* represent cloud resources, their size represent the ECU availability. From *left* to *right* we show deployment strategies with an increasing level of flexibility and therefore increasing cost-saving potential

## Motivating example

Let us consider a real multi-tier cloud application, such as the SAP ERP [24]. This application is composed of two components: an application server and a database server. The application must also satisfy some quality requirements in terms of response time in fulfilling requests. The problem we want to solve is to find the cheapest way to run this application on a spot cloud system while maintaining the quality requirements. To help making this decision we assume to have the following information: (i) a performance model of the application, which can be represented as a queueing network as shown in [24]; (ii) current and historical pricing of the resources that can be rented by the cloud provider; (iii) a quality requirement in terms of constraints on the response time.

For example, assume that, after analyzing the performance model of the application and the expected load, we need VMs with different computational requirements (expressed as Amazon Elastic Computing Units, ECUs) for the application server and the database server. Then, we have a very large decision space on how to deploy them in a cloud infrastructure if we have multiple types of resources characterized by different prices and speeds, such as in Amazon EC2. Figure 1 shows four examples of deployment characterized by an increasing level of deployment complexity. In the first deployment, we make the most intuitive decision, that is to choose the two cheapest resources that can fit the two VMs of the application. In the second deployment we can take advantage of cheap large resources by deploying multiple VMs inside a single large cloud resource. In the third deployment we can take advantage of cheap small resources by replicating application VMs into multiple cloud resources with the help of a load balancer. Finally, in the last deployment we can choose the cheapest VM of any size by combining the two previous deployment approaches, thus obtaining the highest degree of flexibility and cost-saving potential.

In our approach we consider the most complex case and also consider that the deployment decision is not only affected by the size of the cloud resources, as in the deployment example above, but it should take into account also additional real-world characteristics that may affect the overall system performance:

- number of CPUs, since having multiple CPUs does not always correspond to a proportional increase in the system throughput;
- load balancing, since balancing the load among multiple VMs does not always correspond to a proportional increase in the system throughput with respect to using a single resource of the same type;
- availability, since a spot instance has a possibility to be lost and become unavailable for some time.

With respect to existing solutions such as [9, 17, 29], we want to increase the level of accuracy by using *fluid-approximated models* based on differential equations to evaluate the system response time. These systems have been shown in [22] to be able to scale well with respect to the system size and to provide information about the distribution of the response times of the overall system in addition to the average. Moreover, the fluid-approximated models can be easily used with tools like LINE [18, 23] to perform *random environment analysis*. Random environments are stochastic models used to describe events occurring in the environment a system operates in [6]. In our particular situation we model the random environment around spot price fluctuations, so to take into account their effect when computing the mean response time and the response time distribution.

## System model and problem statement

### System model

We begin by considering a model for the system under consideration. The system model we propose is composed of the following two parts: *application* and *resources*. Our goal is to determine the *rental* and *allocation* policies, which consist in the amount of computational resources to be rented from a cloud provider, the mapping of the various application components to these resources, and finally the bid price for each resource.

#### Application

**Fig. 2:**
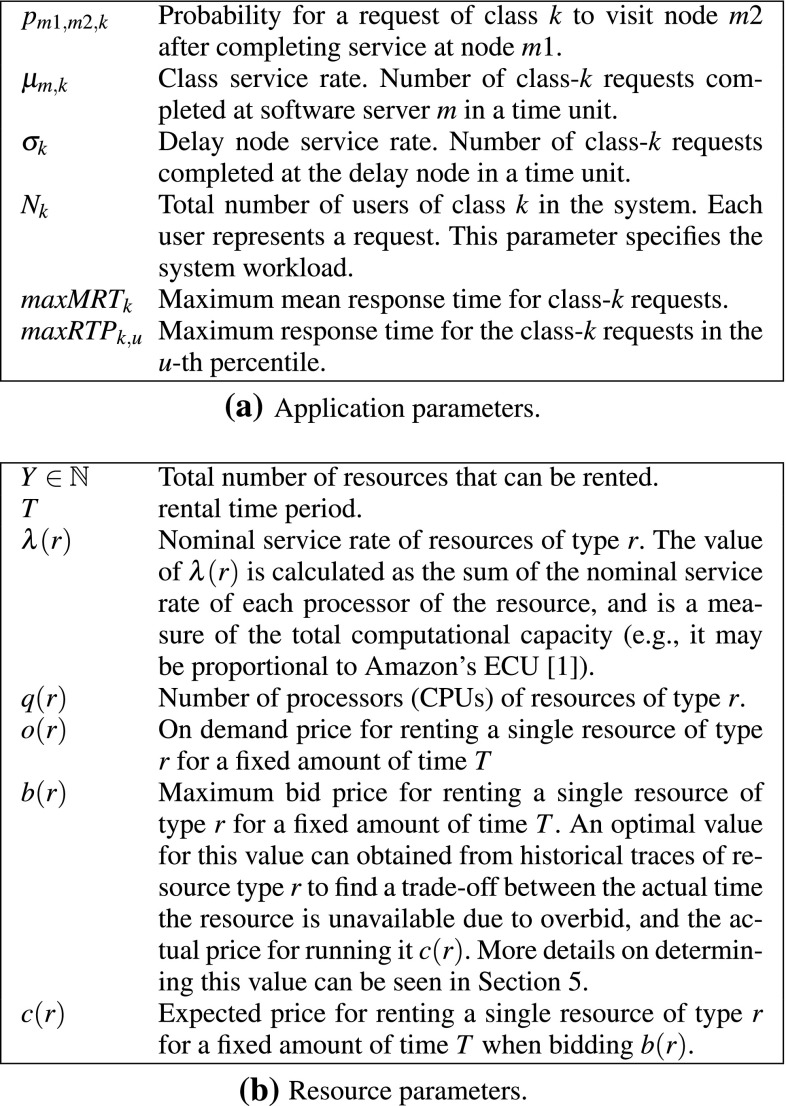
System parameters

We model the application as a closed queueing network $\textit{QN}$ of *M* software servers (representing the application components), a delay node (representing user think time), *K* classes of requests, and a set of constraints on the response time that we defined as Service Level Objective (SLO). A detailed list of application parameters is shown in Fig. 2a.

#### Resources

We consider an environment that has $R+1$ available resource types. Type 0 is a special virtual type used to represent unallocated resources that have zero price and zero rate. Each resource is characterized by a certain rate (processing speed) and a certain number of processors. Moreover, by using historical traces we can also associate to each resource a bid price for obtaining a good compromise between the level of availability, and the actual price that we expect to pay when bidding such bid price. A possible way to estimate a bid price for each resource will be discussed in Sect. 5. More details on the application parameters are described in Fig. 2b.

### Decision variables and problem statement

The system monitors periodically the environment, then it tries to self-adapt the number of cloud resources rented, and the deployment of the software servers on them to optimize the prices and still meet the service requirements. To avoid performance degradation at run-time due to migration and reallocation of software servers, we assume that old resources are deallocated only when the new ones are fully initialized and ready to accept jobs. Based on the considerations above we define our decision variables as follows:

- $t=[t_y]$, $1\le y\le Y$. *Resource assignment vector*: this is a vector that assigns a resource *y* to a resource type $t_y \in \mathbb {N}_{[0,R]}$. From this parameter we also define:
  - $\hat{\lambda }_y:=\lambda ({t_y})$: rate of the resource *y*.
  - $\hat{c}_y=c({t_y})$: price of the resource *y*.
- $D=[d_{m,y}]$, $1\le m\le M$, $1\le y\le Y$. *Allocation matrix*: where $d_{m,y} \in \mathbb {R}_{[ 0,+\infty )}$ assigns part of the rate $\lambda$ of each resource *y* to each software server *m*.

The goal is to decide a resource assignment vector *t* and an allocation matrix *D* that minimize the sum of the prices of all rented resources. A formalization of the optimization problem is the following:

$$\begin{aligned} \text {min}\quad&\sum \limits _{y=1,\ldots ,Y} \hat{c}_y \\ \text {s.t.}\quad&\sum \limits _{m=1,\ldots ,M} d_{m,y} \le \hat{\lambda }_y, \forall y \\&\textit{MRT}_{k}(D) \le \textit{maxMRT}_k, \forall k \\&\textit{RTP}_{u,k}(D) \le \textit{maxRTP}_{u,k}, \forall u, \forall k \end{aligned}$$

The first constraint states that it is not possible to allocate to a resource a rate that is larger than the rate of its resource type. The other constraints state that the calculated mean response time and the response time percentiles should be lower than their respective maximums, where $\textit{MRT}_{k}(D)$ and $\textit{RTP}_{u,k}(D)$ are nonlinear functions to calculate the mean response time and the response time percentiles. These functions have all the decision variables and the system parameters described in Fig. 2 as input, which are omitted to simplify the notation.

## OptiSpot heuristic

### General idea

**Fig. 3:**
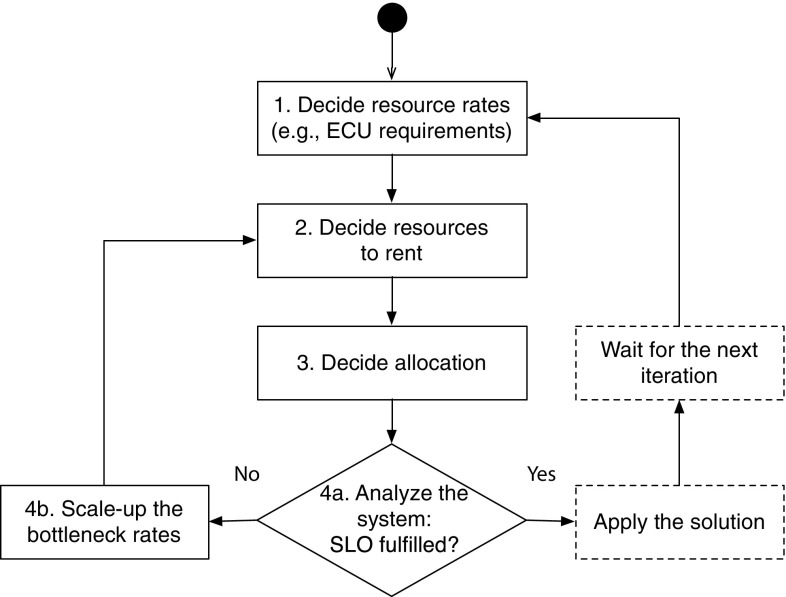
State diagram showing all the steps of the OptiSpot approach. The approach can be seen as an autonomic feedback loop since it adapts the system at periodic interval by using the most updated prediction data available for the resource prices and the application load

The general idea of our approach is to decompose the main problem into simpler subproblems that are solved in an iterative way. Each subproblem obtains its input from the solution of the previous subproblem, as shown in Fig. 3: the numbered blocks in the figure represent the subproblems we solve. Our approach is then repeated at regular intervals as a method of pro-active self-adaptation, or in response to unexpected situations that cause a run-time SLO violation as a method of reactive self-adaptation. A general idea of each subproblem we address is described as follows, while details are given in the next subsections.

1. *Choosing the minimum computational requirements for each application component* In this step we decide the minimum computational requirements in terms of resource rates (e.g., Amazon’s elastic computing units, or simply ECUs) that are needed by each application component to satisfy the quality requirement. At this stage we do not consider the available resources, but we just determine the ECU requirements of the application.

2. *Choosing the resources to rent* In this step we calculate the bidding price that minimizes the cost for each unit of rate (e.g., 1 ECU) and, based on it, we decide which resources to rent. The sum of the ECUs of the rented resources should be large enough to fulfill the ECU requirements of the application decided in the previous step.

3. *Choosing the allocation of the application components to the resources* In this step we decide how to allocate the different application components into the rented resources to minimize the negative effects of allocation (e.g., the reduction in performance due to load balancing, as it happens in the third deployment example in Fig. 1).

4. *Analyzing the overall system and possible scaling-up of bottlenecks* The performance of the overall deployed system is analyzed again taking into account the overhead added by the presence of multiple CPUs and load-balancing. This is also the step in which we consider the effects of the random environment in terms of possibility of losing spot instances and replacing them with on-demand instances in case the chosen bid price is overbid. If this analysis shows that the chosen resources and allocation do not fulfill the quality requirements anymore, the application ECU requirements of the bottleneck software servers are increased to compensate, and new resources/allocations are decided.

### Finding the optimal rate for each software server

In this step we want to find a first approximation of the solution of the global problem by assuming that each software server *m* is deployed on a dedicated hypothetical resource that provides the minimum rate $\hat{\mu }_m$ to process requests such that the SLO constraints are satisfied. In this step we do not consider the characteristics of the real resources (e.g., number of processors, prices, and the random environments information) since a decision on which one to rent will be done in the next steps. The goal of this optimization problem is to decide the minimal rates $\hat{\mu }_m$ that fulfill the constraints on the mean response time and on the response time distribution.

$$\begin{aligned} \text {min}\quad&\sum \limits _{m=1,\ldots ,M} \hat{\mu }_m \\ \text {s.t.}\quad&\textit{MRT}_{k,}(\hat{\mu }) \le \textit{maxMRT}_k, \forall k \\&\textit{RTP}_{u,k}(\hat{\mu }) \le \textit{maxRTP}_{u,k}, \forall u, \forall k \end{aligned}$$

To solve this subproblem we use a greedy algorithm that scales down the rates of all the resources as much as it can until one or more bottleneck resources are found for the class of jobs that is closest to the boundary of the constraints. At this point, the rates of the bottleneck resources are fixed, and the algorithm continues to scale down the remaining rates, until all of them have been fixed in the same way.

**Fig. 4:**
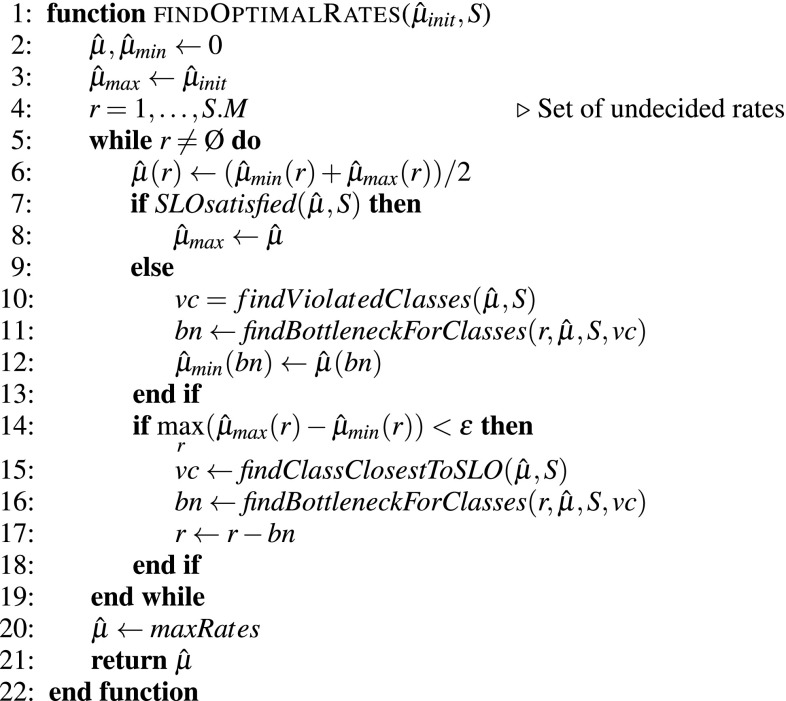
Algorithm for finding the minimum rates $\hat{\mu }$ for each software server

**Fig. 5:**
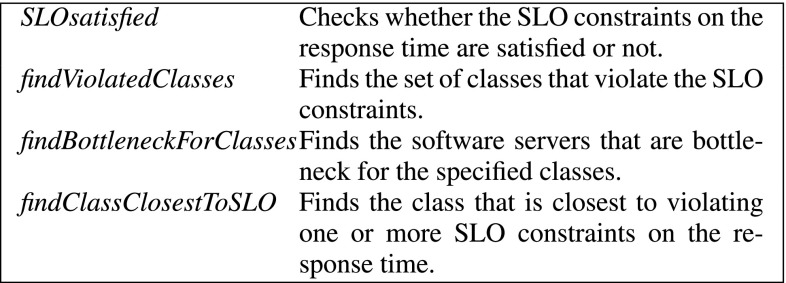
Auxiliary functions that are based on the results of a queuing network evaluation

The pseudocode listing of the algorithm is shown in Fig. 4. The function receives as input an initial set of arbitrarily large feasible rates $\hat{\mu }_{\textit{init}}$, and the system model *S* that contains all the parameters of the application and the resources described in Sect. 3. It returns the optimal rates for each software server as vector $\hat{\mu }$. The variable *r* is initialized as the set of all available resources that can be scaled. Then, all resources are scaled down using a bisection method until the constraints are violated: minimum rates are increased when the constraints are satisfied and the maximum rates are decreased when the constraints are violated. When the minimum and maximum rates are close enough, the current bottleneck resources are removed from *r* and the process continues until *r* is empty. At this point the rate calculated so far is returned as our optimal $\hat{\mu }$. The auxiliary functions used in the algorithm (briefly described in Fig. 5) are directly derived from the evaluation of the queueing network and simple operational analysis laws.

### Finding the real resources to rent

In the previous step we calculated the computational needs in terms of rates of the virtual resources. In this step we want to decide which real resources to rent to provide such computational needs at minimal expense. To make this decision we consider for each real resource *y* a mean price equal to $\hat{c}_y$, that can be obtained from historical traces using the estimation method we discuss in Sect. 5. The goal is to minimize the sum of these costs while ensuring that the rates of all rented resources are large enough to allocate the rates found as the solution of the previous problem.

$$\begin{aligned} \text {min}\quad&\sum \limits _{y=1,\ldots ,Y} \hat{c}_y \\ \text {s.t.}\quad&\sum \limits _{y \in 1,\ldots ,Y} \hat{\lambda }_y \ge \sum \limits _{m \in 1,\ldots ,M} \hat{\mu }_m \end{aligned}$$

This subproblem is a classical integer linear-programming problem (ILP) since the decision variables are integers, and the constraints and the objective functions are linear. This is a well-known NP-hard problem in which we can find an approximate solution using any ILP solver. We implemented a function *findResourcesToRent* to interface with the MATLAB *intlinprog* solver, which accepts the rates of the software servers $\hat{\mu }$ and the system parameters $\textit{S}$ as inputs, and returns the resource assignment vector *t*.

### Finding the allocation of the rate for each software server to the real resources

In this step we want to find a good allocation of the rates found so far for each software server to the rented resources. We can combine the allocation of multiple software servers to a single resource and the replication of a single resource to multiple software server, as in the last example of deployment of Fig. 1. The allocation decision should minimize the overhead due to load balancing by minimizing the number of associations ($a_{m,y}$) between software servers and resources while still ensuring: (i) that each software server obtains at least its minimum rate $\hat{\mu }_m$, (ii) that each rented resource *y* is not providing more than its maximum rate $\hat{\lambda }_y$.

$$\begin{aligned} \text {min}\quad&\sum \limits _{m=1,\ldots ,M} \sum \limits _{y=1,\ldots ,Y} a_{m,y} \\ \text {s.t.}\quad&a_{m,y} = {\left\{ \begin{array}{ll} 1 &{} \text {if }\;d_{m,y} \ne 0 \\ 0 &{} \text {if }\; d_{m,y}=0 \end{array}\right. }, \forall m, \forall y \\&\sum \limits _{y \in Y} d_{m,y} \ge \hat{\mu }_m, \forall m \\&\sum \limits _{m \in M} d_{m,y} \le \lambda _{t_y}, \forall y \end{aligned}$$

To solve this problem we propose an algorithm that finds an approximate allocation by allocating the rates of the software servers having the largest non-allocated rate to the real resources having the largest available capacity in an iterative process until the rates of all software servers have been allocated.

**Fig. 6:**
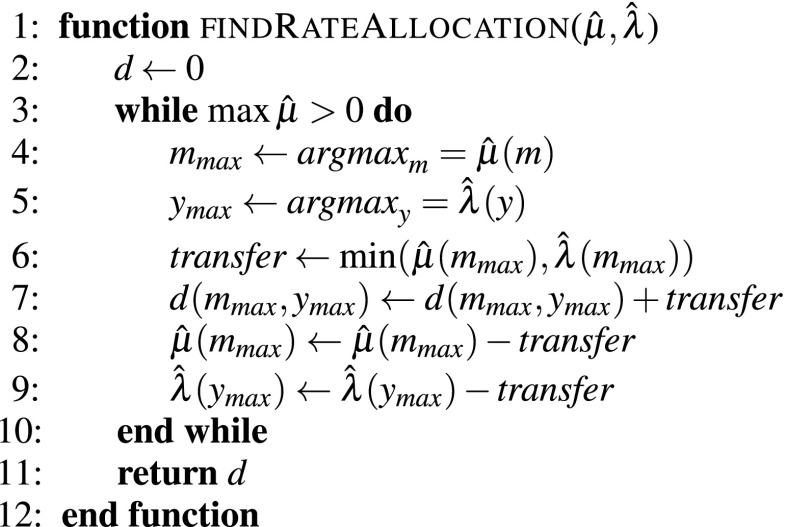
Algorithm for finding the allocation of the rates of the software servers to the real resources

A listing of this algorithm is shown in Fig. 6 as the *findRateAllocation* function. This function takes as input the rates $\hat{\mu }$ we have previously calculated using the *findOptimalRates* function, and the rented resource rates $\hat{\lambda }_y$, which can be derived from the vector of types $t_y$ calculated using the *findResourcesToRent* function with the relation $\hat{\lambda } = \lambda (t_y)$. In each iteration of the algorithm we find the software server with the highest rate $m_\textit{max}$ and the rented resource with the highest rate $y_\textit{max}$. Then, we allocate the maximum rate between the rate of $m_\textit{max}$ and the rate of $y_\textit{max}$ by increasing the corresponding value in the allocation matrix $d_{m_\textit{max},y_\textit{max}}$. To avoid reallocating previously allocated rates, we decrement both the rate of $m_\textit{max}$ and the rate of $y_\textit{max}$ by the allocated value. The process is repeated until all the software servers have zero rate.

### System analysis and scaling-up of the bottleneck server

In this step we check if the SLO constraints still hold when considering the system allocated using the resource assignment vector *t* and the allocation matrix *D* found in the previous steps. In our implementation we use the LINE tool [18] to evaluate the mean response time and the response time percentiles, which considers also real resource parameters such as the number of processors, the load balancing, and the random environment model that describes the possibility for a spot resource to be lost and replaced with an on-demand one when its bid price is overbid.

If, after calculating the response times, the SLO constraints still hold, we can stop here and return the decision variables *t* and *D* calculated so far. These will be used to reconfigure the system and apply the resource rental and allocation decisions.

If the SLO constraints do not hold anymore, it means that the real resource parameters of the proposed allocation had a negative effect on the performance. This can be corrected by identifying one bottleneck server $m_*$ and increasing its rate by a scaling factor $\alpha$, which is calculated proportional to the amount of *constraint violation*. The bottleneck software server is identified as one of the servers that, when scaled-up by $\alpha$, have the best effect in reducing the constraint violation of the SLO. To calculate the SLO constraint violation we use the following method. Given a set of *i* constraints rewritten in the form $V<0$, where $V=[v_i]$, we define the SLO constraint violation as the maximum value in *V*. A positive constraint violation means that at least one SLO constraint has been violated.

**Fig. 7:**
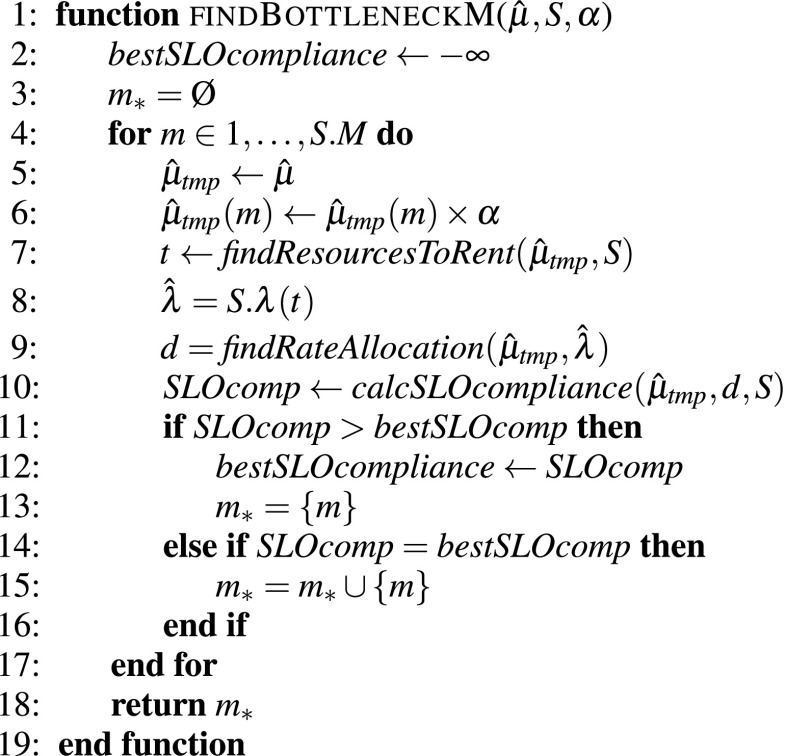
Algorithm for finding the bottleneck software servers

Finally, to actually determine bottleneck software servers $m_*$ we propose the *findBottleneckM* function, which is shown in Fig. 7. This function iterates all the software servers, trying to scale each one up by $\alpha$ and saving the information of the software servers $m_*$ that result in the best reduction of constraints violation. The algorithm then simply recalculates the new resource allocations that would be needed when scaling-up the rate of each software server. Once the bottleneck software servers have been found, we just scale their rate up by $\alpha$ and go back to recalculate the real resources to rent.

### Convergence of the approach

In this concluding section we give some final remarks on the convergence of each step of our approach.

The problem of finding the optimal rate (step 1) has a guaranteed convergence since it uses the bisection method for fixing the rate of the *M* resources associated to the software server. The maximum number of queueing network evaluations needed is $O\big (M\times \textit{log}_2(\textit{max}(\hat{\mu }_{\textit{init}}))\big )$, where *M* is the number of software servers and $\hat{\mu }_{\textit{init}}$ is the vector containing the initial random feasible rates that are given as input to the *findOptimalRates* function.

The problem of finding the real resources to rent (step 2) is NP-hard and solved using an approximated ILP solver. The convergence and the complexity of this step therefore depends on the ILP solver used and its parameters. In this step no queueing network evaluations are performed.

The problem of finding the allocation (step 3) has a guaranteed converge since at each iteration some rate is transferred from the software server with the maximum unallocated rate to the rented resource with maximum rate availability. The maximum number of rate transfers happens when all the *M* software servers are transferred to all the *Y* rented resources, therefore the number of iterations of this step is $O(M \times Y)$. Similarly to step 2, this step does not perform any queueing network evaluation during its iterations.

Finally, in the last step it is possible that the final solution computed is not feasible (i.e., it violates the constraints). In this case we need to search for bottleneck servers and scale them up by a factor $\alpha$. The algorithm to find the bottlenecks tries to scale-up all the software servers one by one, thus resulting in *O*(*M*) queueing network evaluations for each search. Each search guarantees that the bottleneck resources speed is increased, thus progressively reducing the violation of the constraints until an optimal solution is found. In some limit situations it is possible that an increase in the rate of a bottleneck resource does not reduce the violation of the constraints, which would prevent the convergence of our approach. These limit cases happen when the contribution to the response time added by the load balancing, the multiple number of processors, and the random environment is too large to be compensated by an increase in rate. Examples of these limit situations are cases with very low resource rates or in which bid prices are continuously overbid and underbid. In our experiments based on real data we did not experience any of such limit cases, which leads us to think they are contrived examples.

## Bid price and random environment

In the previous section we have shown the OptiSpot heuristic to decide how many resources to rent from a cloud infrastructure and how to map the application components to them. The approach requires to have a resource model, as explained in Sect. 3. In particular, it is important to determine the value of the bid price *b*(*r*) for each resource *r*. From *b* it is possible to derive the expected cost *c*(*r*) and other information regarding the possibility to lose the resource, which are needed by our heuristic to evaluate the QN. To simplify the notation, in the remainder of this section we omit the resource type index *r* since we are always referring to a single generic resource type.

### Determining the parameterized resource model

To determine the parameterized resource model in our case study, we use Amazon EC2 historical spot price traces for each type of resources that are available as text files in [10]. Each line of each trace contains the timestamp and the updated market price for such resource. By analyzing the trace, given an arbitrary bid price *b* (which is our main decision parameter), we can directly estimate the following three functions:

- *overbidTime(b)* mean time before the bid price *b* is overbid (i.e., an active spot instance is reclaimed by the cloud provider).
- *underbidTime(b)* mean time before the bid price *b* is underbid (i.e., a previously reclaimed spot instance is available again).
- *spotCost(b)* average cost for an active spot instance, when bidding *b*. The difference between this cost and *c*(*r*) is that the former only considers the use of spot instances, while the latter considers the possibility for a spot instance to become an on-demand instance when the bid price is overbid, which is the actual cost incurred by the user.
- *A(b)* expected availability of the resource when bidding *b*. This function estimates the percentage of time the resource is able to process requests.

We assume that each resource can be rented as a spot instance or as an on-demand instance, or as both, depending on the situation. For example, if a resource is running as a spot resource and the bid price is overbid, such resource will be eventually lost (after a termination notice grace period) and replaced with an on-demand resource until the spot price becomes lower than the bid price. In our model the transition between spot and on-demand happens according to the *overbidTime(b)* and *underbidTime(b)* functions, and some additional fixed parameters that depend on the type of resource rented. These additional parameters are explained in Table 1.

**Table 1:** Fixed Amazon EC2 parameters for each resource *r*

*termNoticeTime*	Time between a spot instance is overbid and its termination (this is the advance termination notice service offered by Amazon EC2)
*odStartupTime*	Time needed to start this resource in on-demand mode
*spotStartupTime*	Time needed to start this resource in spot mode

**Fig. 8:**
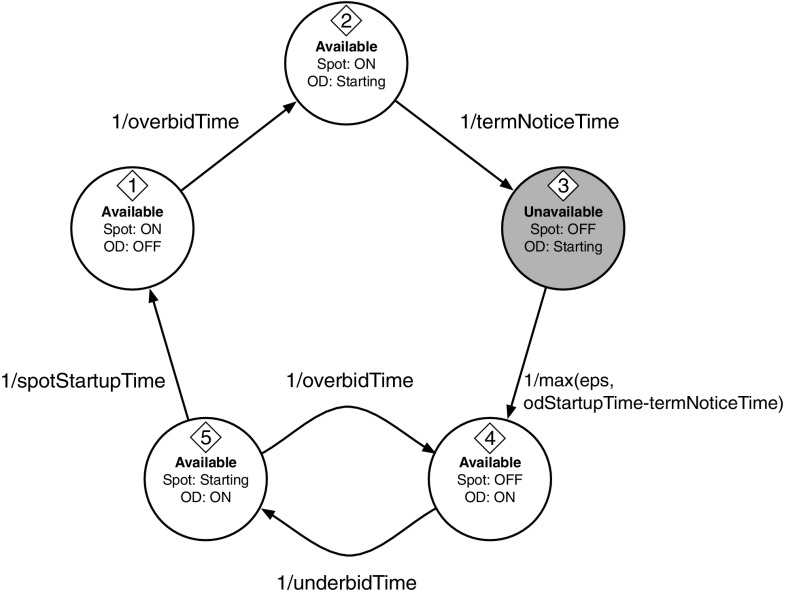
CTMC representing the different states of each resource. Once the optimum bid *b* is determined, it also represent the random environment of the system. “Available” states are the states in which the resource is available. The “Unavailable” (*red*) state is a transition state in which a spot resource is lost and the replacement on-demand one has not been started yet (Color figure online)

The states of each resource *r* and the transition frequency among states are represented as the Continuous-Time Markov Chain (CTMC) in Fig. 8:

- *State 1* The resource is available as a spot instance. Spot price is paid.
- *Transition 1 to 2* The bid price has been overbid, the spot instance receives a termination notice, and an on-demand instance is scheduled to start.
- *State 2* The resource is available as a spot instance (although it has received the termination notice) and an on-demand instance is starting. Both spot and on-demand prices are paid.
- *Transition 2 to 3* The termination notice is expired and therefore the spot instance is no longer available.
- *State 3* The resource is not available, it is being started as an on-demand instance, but it is not ready yet to process requests. On-demand price is paid.
- *Transition 3 to 4* The on-demand instance is now ready to receive requests. This transition can happen instantaneously in the case the time needed to start the on-demand VM (*odStartupTime*) is not higher than the termination notice time (*termNoticeTime*). To avoid the possibility of having a non-positive period in the CTMC for this transition, we force a lower bound equal to *eps* (the smallest positive number that can be represented).
- *State 4* The resource is available as an on-demand instance. On-demand price is paid.
- *Transition 4 to 5* The bid price has been underbid, so it is possible to start a spot instance again.
- *State 5* The resource is available as an on-demand instance, although a spot instance is currently starting. Both spot price and on-demand price are paid.
- *Transition 5 to 1* The spot instance is now ready to receive requests and the on-demand instance is terminated.
- *Transition 5 to 4* The spot instance has been overbid before being fully started. So it is immediately terminated since an on-demand instance is still active.

For a given bid price *b*, we calculate the stationary distribution of the CTMC described above as $\Pi _x(b)$, for each state $x \in 1,\ldots ,5$. From the stationary distribution we can then calculate the resource availability and the expected cost, expressed as functions of *b*:

$$\begin{aligned} A(b)\,=\,&1-\Pi _3(b) \\ c(b)\,=\,&\textit{spotCost}(b) \times (\Pi _1(b) + \Pi _2(b) + \Pi _5(b)) + \\&+ \,o \times (1-\Pi _1(b)) \end{aligned}$$

The availability *A*(*b*) is calculated as the probability for not being in State 3, which is the only state in which the resource is not serving requests. The actual hourly cost *c*(*b*) is calculated as the sum of the costs for having a spot instance active plus the sum of the costs for having an on-demand instance active (*o*, defined in Sect. 3, is the fixed on-demand price for the resource).

### Determining the bid price

Once we have our parameterized resource model, we can determine the bid price that minimizes the following objective function:

$$\begin{aligned} \text {min}_b\quad \frac{c(b)}{A(b) \times \lambda } \end{aligned}$$

The objective function measures the actual price for each unit of resource rate ($\lambda$), which is scaled proportionally to the time in which the resource is available (*A*(*b*)). If the value of the objective function, once maximized, is higher then $o/\lambda$, then the resource is always cheaper in on-demand mode. However, from the traces we have analyzed, we have never encountered the situation in which an on-demand instance is always better than the spot/on-demand switching scheme we propose. It is important to notice that, although we propose to estimate the bid price from historical traces (e.g., hours, days, or even months), this might not always be true since a past fluctuation is not necessarily correlated to a future one. Our bidding estimation approach is very conservative regarding this point since in the worst case scenario the system reverts to on-demand resources until a new more updated estimation is computed. This bid estimation approach is orthogonal with respect to the OptiSpot heuristic, and, based on the user needs, can be replaced with some alternative bidding approaches such us the ones we will discuss in Sect. 8.

### Determining the random environment

Once we have found an optimal value for the bid price *b*, we can instantiate the CTMC in Fig. 8 and use it as our random environment representation. States 1, 2, 4, 5 represent a resource in a normal working situation, therefore the QN will be evaluated using standard rates for the resource. State 3 represents the situation in which the resource is not able to process requests, and it corresponds to a QN with zero rates for that resource, meaning that all the requests will be put in the queue until the resource exits State 3. These enqueued jobs are expected to worsen both the mean response time and the response time distribution. Our QN solver is able to support CTMC representations for the random environment and therefore our heuristic will take into account the effects of the possibility to lose a resource due to price fluctuations when calculating an optimal deployment for an application.

## Evaluation setting

The purpose of our evaluation is to give an overview of the behavior of our approach when applied to queueing network models based on real data. In particular, we use public application data measurements from a real SAP ERP study from [24]. For the resources model we use historical traces of spot prices of Amazon EC2 (provided directly from Amazon) that can be downloaded from [10] and cover a 3-month period up to January 2016. Finally, we instantiate our problem using the generic non-linear solver provided by MATLAB to compare it with our approach. The remainder of this section discusses more in detail the hardware, software, and application models we used to perform our experiments. The results will be presented in Sect. 7.

### Hardware and software

We performed our experiments using a 2.5 GHz Intel Core i7 quad-core processor with 16 GB of RAM running OS X 10.11.1 and MATLAB R2015b. We also used LINE 0.7.1 [18] to predict the response times of our queueing network, and we implemented all the functions described in Sect. 4 as MATLAB functions. To allow the evaluation of the effect of allocating the VM of a software server to multiple resources (i.e., replicating it), we have implemented a function to split the nodes of the queueing network according to their allocation to real resources (*allocateQN*, *splitStation*); moreover, we have implemented an alternative solution to the problem using MATLAB *fmincom* nonlinear solver configured with an interior point algorithm, which we refer as the *exact approach*. This alternative solution considers exactly the same model we solve with our heuristic, but without any particular optimization that can guide the algorithm toward the proper solution. We have chosen this generic solution due to the limited availability of existing approaches that adopt our model formulation.

For the sake of simplicity we omit accurate descriptions of these functions, but they can be downloaded, with all the other MATLAB code we have implemented, from [10]. The provided code can be used to repeat our experiments or to interface it with the run-time monitoring and adaptation module of a cloud system to perform follow-up research on the full autonomic adaptation loop.

### Application model

We use an application model based on previous measurements of an industrial ERP application, SAP ERP. The data of this model and its queueing network representation have been derived from [24] (page 5, Table 2). The application model is represented as a queueing network with exponentially distributed service times, $M=2$ software servers (representing respectively the CPU of the application server and the CPU of the database server), a delay node representing the user think time, and $K=3$ classes of requests, which are:

- *dialog step* requests: process and update data on the client-side through the graphical user interface;
- *update* requests: higher priority asynchronous update requests that may be triggered by a dialog step request;
- *update2* requests: lower priority asynchronous update requests that may be triggered by a dialog step request.

**Fig. 9:**
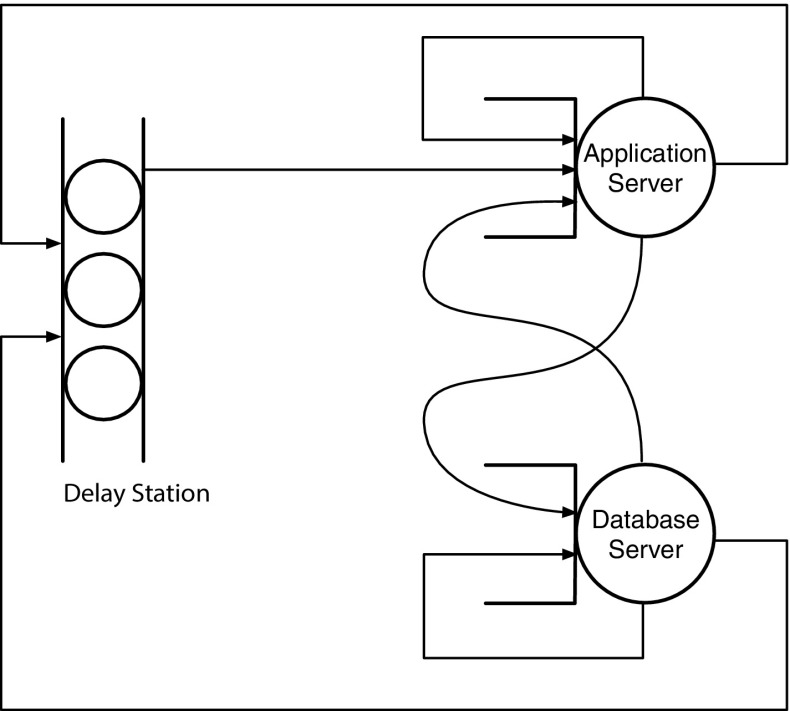
Queueing network representation of the SAP ERP application. The delay station models the user think time, and it is represented as a station with infinite servers. The application server and the database server are represented as regular queues

The SAP ERP application included additional types of requests, but in the study we are using as reference they were ignored because of their negligible effects on the response times. From the paper we used the information of the service demands, number of users, and number of transactions at each software server and for each class. From this information, we were able to determine a value for the class services rates ($\mu _{m,k}$) and the routing probabilities ($p_{i,j,k}$). A graphical representation of the queueing network is depicted in Fig. 9. In [24] the authors just give an estimation of the overall service demands of the database server, without distinguish the classes of requests. To overcome this problem we assume that the database CPU demand is distributed across the different classes proportionally to the number of users for such classes. The data we have obtained for the class service rate can be seen in Table 2, which is calculated as the inverse of the service demand. Additional application parameters are the following:

- $N_\textit{dia}$ (number of users that issue dialog step requests) is arbitrary, but $N_\textit{upd}$ (number of users that issue update requests) and $N_\textit{upd2}$ (number of users that issue update2 requests) are assumed dependent on it, as explained in Sect. 3.4.1 of [24]. Therefore we consider $N_\textit{upd}=0.2652 \times N_\textit{dia}$ and $N_\textit{upd2}=0.06657 \times N_\textit{dia}$.
- $\sigma _k$ is 0.0001 for all service classes, since we assume an average think time of 10 s for each class of users in the system.

**Table 2:** SAP ERP parameters

Server/class	Service demand (ms)	Service rate $\mu _{m,k}$ (req/ms)
AS dialog step	119.82	0.008346
AS update1	47.92	0.02087
AS update2	32.98	0.03032
DB dialog step	4.541	0.2202
DB update1	1.205	0.8299
DB update2	0.3043	3.286

**Fig. 10:**
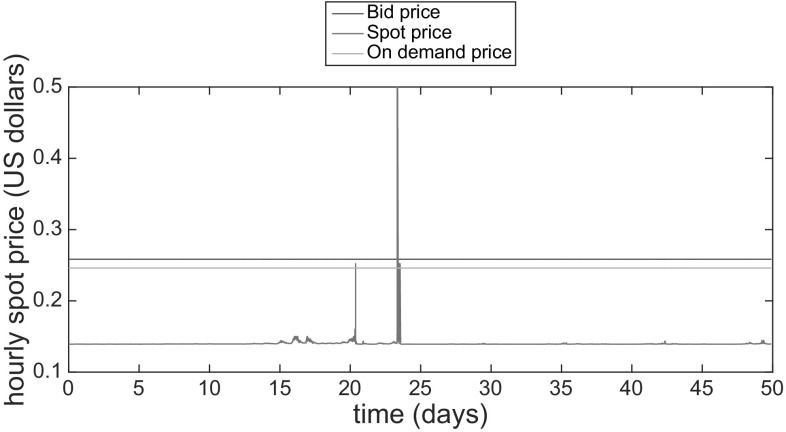
Example of Amazon EC2 spot instance trace for a Windows m4.large VM of the us-east region. The trace shows the spot price fluctuations for 50 days starting on October 10, 2015 and compares them with the on-demand and estimated bid price. It is possible to notice that the overbid events happen very rarely (Color figure online)

### Resource model

To determine the resource model we use Amazon EC2 historical spot price traces for each type of resources that are available as text files in [10]. An example of 100 h trace is shown in Fig. 10.

To determine the resource parameters introduced in Sect. 3 we used the estimation approach described in Sect. 5. In particular, we use the fixed parameters shown in Table 3, which contain the time needed to start on-demand and spot resources (*odStartupTime* and *spotStartupTime*), as measured and reported in previous work [20]; and a fixed advance termination notice time as stated by the current Amazon EC2 policy, which is generated in case of overbid (*termNoticeTime*). All the other parameters of the resource model are shown in Table 4: the first column contains the resource type, the region, and the operating system; columns 2–4 show the resources characteristics and on-demand prices, as advertised by Amazon EC2; column 5 shows the estimated optimal bid price; and finally, columns 6–9 show the other parameters that are functions of the bid price.

From Tables 3 and 4 we can see the following particularities:

- *Linux instances have 100 % availability* This happens because the termination notice is higher than the time needed to start an on-demand resource, therefore the resource will never be in the unavailable state (state 3 of the CTMC in Fig. 8).
- *Some resources have an infinite overbid time* This happens when the current bid price has never been overbid in historical traces. This also results in a 100% availability since the spot instance is assumed to never terminate.
- *Some resources have a bid price that is slightly higher than the on-demand price* This is intentional since we want to avoid the situation in which a resource switches too often between spot mode and on-demand mode, which would cause a decrease in the availability and consequently in the amount of processed requests per price paid.
- *The availability level is quite high for all the resources* This is a result of the method we used to calculate the optimal bid price, which tries to find the best trade-off between the actual price paid and the availability. Another reason for the high value is the fact that the time during which a resource is unavailable is less than the time needed to start the new on-demand resource since the new on-demand resource is started proactively after the spot instance termination notice from Amazon EC2 is received.

**Table 3:** Values of fixed parameters for each resource *r*

Parameter	Linux VMs (s)	Windows VMs (s)
*odStartupTime*	97	810
*spotStartupTime*	557	1270
*termNoticeTime*	120	120

**Table 4:** Amazon EC2 resource parameters and prices in US$

Resource	Rate ECU	CPUs	On-dem. price ($/h)	Bid price ($/h)	Actual price ($/h)	overbid Time (h)	underbid Time (h)	Avail. (%)
*r*	$$\lambda (r)$$	*q*(*r*)	*o*(*r*)	*b*(*r*)	*c*(*r*)			*A*(*r*)
m3.medium (us-east/Linux)	3	1	0.067	0.0704	0.0104	4416	0.0814	100
m3.large (us-east/Linux)	6.5	2	0.133	0.1463	0.0235	155	0.4408	100
m3.xlarge (us-east/Linux)	13	4	0.266	0.266	0.0391	252	0.1901	100
m3.2xlarge (us-east/Linux)	26	8	0.532	0.532	0.1029	$$\inf $$	0	100
m4.large (us-east/Linux)	6.5	2	0.12	0.132	0.0278	65	0.8569	100
m4.xlarge (us-east/Linux)	13	4	0.239	0.2629	0.0438	145	4.3378	100
m4.2xlarge (us-east/Linux)	26	8	0.479	0.5269	0.0984	$$\inf $$	0	100
m3.medium (us-east/Windows)	3	1	0.13	0.13	0.0591	$$\inf $$	0	100
m3.large (us-east/Windows)	6.5	2	0.259	0.272	0.1176	420	0.1726	99.95
m3.xlarge (us-east/Windows)	13	4	0.518	0.518	0.1336	1262	0.0787	99.98
m3.2xlarge (us-east/Windows)	26	8	1.036	1.036	0.2767	2208	0.1076	99.99
m4.large (us-east/Windows)	6.5	2	0.246	0.2583	0.1409	140	0.0956	99.86
m4.xlarge (us-east/Windows)	13	4	0.491	0.5156	0.28	676	3.2325	99.97
m4.2xlarge (us-east/Windows)	26	8	0.983	1.0322	0.5559	$$\inf $$	0	100
m3.medium (eu-west/Windows)	3	1	0.129	0.129	0.0722	3312	0.0711	99.99
m3.large (eu-west/Windows)	6.5	2	0.258	0.258	0.1432	2208	0.176	99.99
m3.xlarge (eu-west/Windows)	13	4	0.517	0.5429	0.2288	471	1.9457	99.96
m3.2xlarge (eu-west/Windows)	26	8	1.033	1.0846	0.5436	$$\inf $$	0	100
m4.large (eu-west/Windows)	6.5	2	0.244	0.2562	0.1269	662	0.5042	99.97
m4.xlarge (eu-west/Windows)	13	4	0.488	0.488	0.2561	212	2.0831	99.91
m4.2xlarge (eu-west/Windows)	26	8	0.976	0.976	0.5061	2207	1.6699	99.99

Based on traces between 10/10/2015 and 10/01/2016

Summarizing, we run our analysis on general-purpose *m*3 and *m*4 Linux and Windows instances of the *eu-west* and *us-east* Amazon EC2 regions. We represent the random environment of the system as the CTMC in Fig. 8, which expresses the possible states of each resource: available (when it is able to process requests) and unavailable (when it is not able to process requests because a lost spot-instance is being recovered using an on-demand instance). Our code for generating our resource model is contained in the classes *Survival* and *Resources*, available in [10].

Since the application model has the rates expressed as requests/sec using a reference system that is not expressed in ECU, we have found the conversion rate 1 ECU = 65.1 requests/sec by choosing a rate to the SAP ERP application such that the response time with 1 ECU is equal to the response time measured in [24].

## Experiments and results

**Fig. 11:**
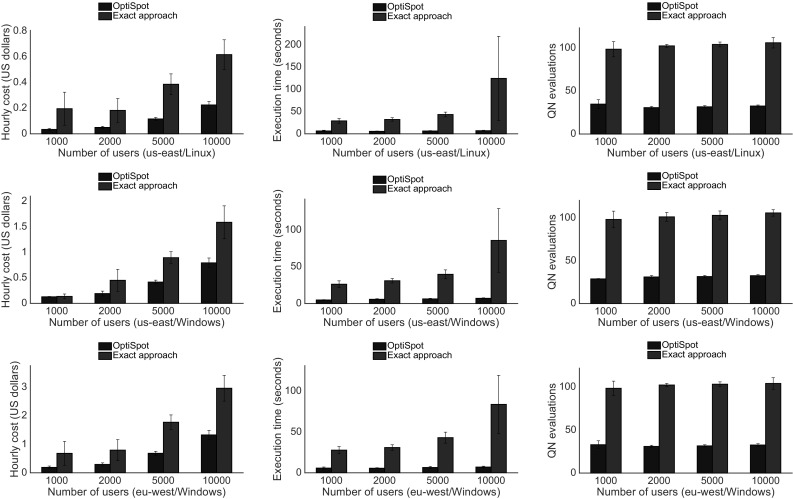
Experiment results when varying the number of users (Color figure online)

**Fig. 12:**
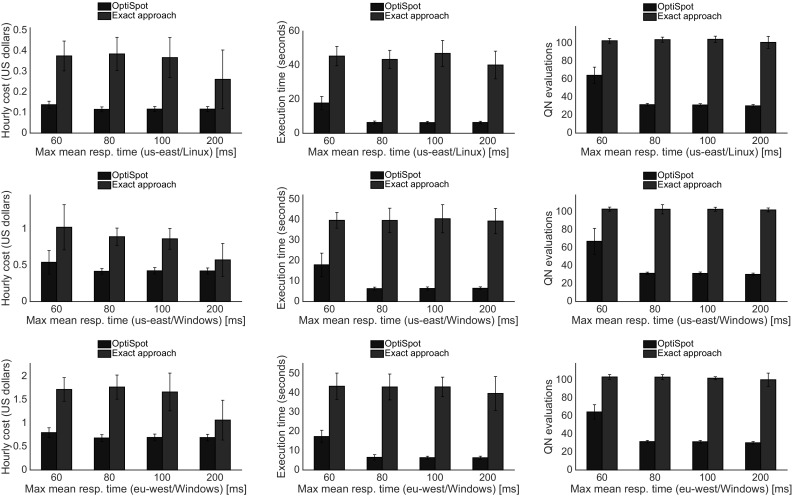
Experiment results when varying SLO. The SLO is a maximum limit on the mean value of the response time calculated for a rental time period *T* (Color figure online)

**Fig. 13:**
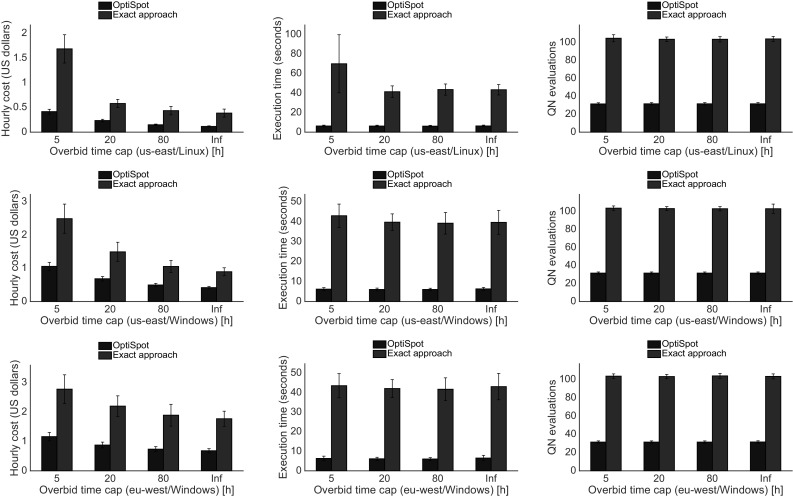
Experiment results when the overbid time is capped and the minimum underbid time is fixed (Color figure online)

We evaluate the real SAP ERP application described in the previous section under different scenarios characterized by a variable number of users to analyze the scalability; with different SLOs, to analyze the behavior in more challenging situations; and finally with capped overbid time and fixed minimum underbid time, to analyze the effects of the random environment when the chances for a resource to be overbid is increased. Each experiment has been repeated in two different Amazon EC2 regions (eu-west and us-east) and with different Operating Systems (Linux and Windows). In each scenario we measure the expected hourly price of the resources, the time needed to compute the solution on our system, and the number of queueing network evaluations. We repeat every evaluation 30 times with a different search starting point to ensure statistical confidence of the results and to show the standard deviation bars in each plot.

### Varying users

In this experiment we vary the number of dialog users in the system from 1000 to 10,000. We fix a SLO that consists of a maximum average response time of 80 ms and a maximum 80th percentile of the response time distribution equal to 320 ms. By looking at Fig. 11 we can see that both our approach and the exact one tend to have a price that grows proportionally with the number of users across different zones and OSes. The total number of queueing network evaluations tends to be similar for different number of users: in the case of our heuristic we have the convergence at around 30 evaluations, while in the exact approach we often reach the cap of 100 evaluations that has been set to keep the comparison fair. Interestingly, we can see that the execution time is not proportional to the number of evaluations. The reason for this is that the actual time for one queueing network evaluation is proportional to each assignment of software server to a cloud resource. Our heuristic intentionally reduces the level of fragmentation of the assignments to the resources to reduce the overhead due to load balancing, while the exact approach explores too many alternatives that include situations with a high level of fragmentation (i.e., software servers are assigned to a high number of rented resources).

### Varying SLO

In this set of experiments we show what is the effect of different SLOs constraints on the total hourly price of the system. We consider a maximum response time $\textit{maxMRT}$ that varies from 60 to 200 ms, a value of $\textit{maxRTP}_{80}=4\times \textit{maxMRT}$ and 5000 dialog users. The results in Fig. 12 show that there is an increase in price when the SLOs are more challenging for both algorithms; however, our approach is still better than the exact one for every different SLO we have considered. From this experiment we can also notice that when the situation becomes more difficult (stricter SLO), we have a significant increase in the number of evaluations and in the time to find the solution. The explanation is that when the SLO is too strict our heuristic requires an increasing number of scaling-up steps.

### Varying overbid time cap

In this experiment we want to see how OptiSpot behaves in stressful situations in which the overbid and underbid times are much worse than the ones predicted from the real historical traces. To do this we fix a maximum cap to the overbid time that varies from 5 to 80 h and a fixed minimum value for the underbid time equal to 5 h. This means that the spot price has a higher chance to be overbid and a lower chance to be underbid when compared to the non-capped experiments. The other parameters we have chosen are the reference ones: 5000 dialog users, $\textit{maxMRT}=80\, \mathrm {ms}$, and $\textit{maxRTP}_{80}=320\, \mathrm {ms}$. In Fig. 13 we observe that, when the overbid time is minimum, the cost is maximum, while when the overbid time increases, the cost becomes lower and converges to a value that is similar to our non-capped experiments (labeled as “$\inf$” cap in the Fig. 13). The explanation for this is that a small overbid time means that the time needed for the bid price to be overbid is small and therefore resources may become unavailable and switch to the more expensive on-demand instances more frequently. If we look at the number of QN evaluations and at the time needed to compute the solutions, the results are similar, which means that our heuristic is able to dead efficiently with this situation.

Since the overbid and underbid times used in these experiments are much worse than the ones measured from the real Amazon EC2 traces, we can conclude that our approach is able to support application deployment decisions and to behave better than the exact approach even in scenarios that are much more extreme than the ones considered in our case study.

### Discussion

In this analysis we have seen that our approach is able to outperform an exact algorithm that is based on the MATLAB fmincon interior-point solver. The reason for this result is that our heuristic is able to choose the resources with the best price/ECU ratio and to allocate the application components in such a way that they are not fragmented among cloud resources unless the number of resources is smaller than the number of components. If the number of resources is small, such as in the case of 1000 users, there is minimal difference between our approach and the exact one. As the number of users increases, or the SLO becomes more restrictive, we need more cloud resources to fulfill the SLO. When the number of resources becomes larger than the number of application VMs, the exact approach is not able to choose the correct size of the resources since it tries to resize the partitions of multiple resources, leading to oscillations and slow convergence. The high number of partitions also results in a higher time to evaluate the fluid-approximated queueing network, which ultimately results in large total execution times. Unfortunately, due to the limitations of fmincon we could not express a fitness function that was good enough for the exact approach to converge in every situation. However, in situations in which we observed convergence, the computation of the result was always significantly slower.

## Related work

In the previous sections we have seen that the main idea of this paper is to combine cost-aware cloud resources provisioning and application mapping into a synergistic autonomic solution that takes into account performance requirements and environmental random factors such as the prices fluctuation of cloud resources and user load.

The problem of exploiting different cloud pricing methods such as spot instances has been studied in literature since Amazon introduced the service in 2009. Some works (e.g., [13, 14, 28, 30, 31]) focus on understanding the price dynamics to generate price predictions that can be used to make cost-effective provisioning decision. Other works (e.g., [2, 29, 32]) focus on providing bidding strategies that are specific for spot instances. Our work does not claim to replace or be better these existing methods for forecasting resource prices and decide what to bid, but to complement them. In fact in our evaluation we simply assume that bid prices observed so far tend to repeat in the future; however, in situations when our assumption on the future bid prices is not true, we can seamlessly benefit from the alternative methods cited above without the need to change our heuristic.

Some different works such as [8, 15, 27] give tools that encourage the use of spot resources by increasing their reliability in case of outbid using recovery techniques based on checkpointing or replication. In our work we are aware of the system reliability thanks to the use of random environment for representing the possibility to lose spot resources; however, the possibility to use reliability-increasing techniques is also orthogonal to our approach and a combined one may result in additional savings in the total price for renting resources.

Finally, research on service placement and load allocation has been specialized to take into account spot pricing models and the possibility to lose resources unexpectedly [4, 5, 9, 12, 16, 17, 19, 33, 34]. With respect to these works we also solve the allocation problem in such a way to minimize the costs while maintaining the desired service level. Our new contribution is that we adopt fluid-approximated performance models [22], which can calculate response time distributions quickly enough to be used at run-time. We also use a random environment model [6] to represent the effects of external events to the system, which for now is limited to price fluctuations, but that can be easily extended to other events expressible as stochastic models. Finally, in our model we also consider the effects of having multiple CPUs in cloud resources (as it is the case for Amazon EC2) and the overhead due to load balancing in case of placement decisions that require resource replication.

## Conclusions

In this paper we have presented OptiSpot, a cost-aware approach to support run-time decisions for provisioning cloud resources and allocating application components among them. The benefit of OptiSpot is that it is able to approximate a very complex problem using simple greedy algorithms that are lightweight enough to be used at run-time to support pro-active and reactive system adaptation. Moreover, we have shown a possible way to optimize the bid price that makes use of a Markov chain representation of the system. We then used the same Markov chain, instrumented with an optimal bid price, to have a representation of random environmental parameters such as the possibility for spot resources to be lost and replaced with on-demand ones. The random environment is used by OptiSpot to predict system performance and make deployment decisions even when price fluctuations modify the resources and the system ability to process requests during state transitions.

The decisions produced by our approach are designed to be used to trigger allocation, deallocation, migration, and replication actions on one or more cloud infrastructures. In our model we assumed that these actions do not affect performance since we consider to keep the system running while they occur; however, this might not be true in every system.

Some future work we have in mind is to introduce in our models and heuristics the possibility to take into account possible overhead in terms of time, performance, and cost that can arise when actually performing adaptation actions on a real system. We also intend to investigate how the approach behaves in presence of different cloud platforms (e.g., federated clouds [21]), services, and alternative ways of expressing the SLOs. Finally, another possible follow-up work is to extend our approach to decentralized cloud systems to improve the scalability and resistance to dynamism, which may contribute to support new emerging cloud paradigms such as volunteer clouds [26] and edge clouds [7, 25].
